# *Vibrio metschnikovii* Pneumonia

**DOI:** 10.3201/eid1110.050177

**Published:** 2005-10

**Authors:** Frédéric Wallet, Mickaël Tachon, Saad Nseir, René J. Courcol, Micheline Roussel-Delvallez

**Affiliations:** * Lille University Medical Center, Lille, France

**Keywords:** Immunocompromised, pneumonia, Vibrio metschnikovii, letter

**To the Editor:** *Vibrio metschnikovii* is a gram-negative, oxidase-negative bacillus. This species was isolated in 1981 from blood culture of an 82-year-old diabetic woman with cholecystitis (*1*). It was previously isolated from river water, clams, oysters (*2*), fish (*3*), and birds that died of a choleralike illness (*4*). We report isolation of *V. metschnikovii* in bronchial aspirate from a patient with pneumonia.

A 63-year-old man was admitted to the intensive care unit (ICU) of A. Calmette hospital in Lille, France, for acute respiratory failure related to community-acquired pneumonia. The patient had a history of chronic obstructive pulmonary disease with a forced expiratory volume of 820 mL in 1 s (32% of predictive value); he was treated with oral salmeterol, terbutaline, and prednisolone (40 mg/day). He was HIV negative. On ICU admission, he had the following values: respiratory rate 30/min, temperature 39°C, pulse rate 140/min, blood pressure 140/90 mm Hg, Glasgow coma score 15, leukocyte count 13.7 × 10^9^/L, hemoglobin level 10.2 g/dL, procalcitonin level 22 ng/mL, and C-reactive protein level 73 mg/L. A chest radiograph showed diffuse bilateral infiltrates. Analysis of arterial blood gases with 6 L of oxygen/min showed respiratory acidosis: pH 7.30, pCO_2_ 59 mm Hg, pO_2_ 78 mm Hg, HCO_3_ 23 mmol/L, O_2_ saturation 94%. Other laboratory test results were normal. After noninvasive ventilation failed, the patient was immediately intubated and mechanically ventilated.

Blood cultures and bronchial aspirate samples were obtained before initiating treatment with antimicrobial drugs. The patient was treated with amoxicillin/clavulanic acid and ciprofloxacin. Blood cultures showed negative results. Microscopic examination of the bronchial aspirate showed no squamous epithelial cells, a few gram-negative bacilli, leukocytes, and many ciliated bronchial cells. The presence of ciliated cells was the best indicator that secretions originated from the lower respiratory tract. A urinary antigenic test result for *Legionella* spp. was negative. Quantitative culture of the bronchial aspirate on bromocresol purple agar, blood agar (grown in an atmosphere of 5% CO_2_), and chocolate agar plates yielded *V. metschnikovii* (10^7^ CFU/mL) and nonhemolytic streptococci (10^5^ CFU/mL) as the oropharyngeal flora. These streptococci (gram-positive, catalase-negative) were not considered to be the pathogenic agent.

The strain of *V. metschnikovii* isolated was a gram-negative, curved rod. This facultative aeroanaerobic bacillus formed opaque colonies (diameter 3 mm) on blood agar in 24 h and showed complete hemolysis. It was catalase positive, oxidase negative, and did not reduce nitrate to nitrite. This strain was identified as *V. metschnikovii* with an ID GBN Vitek 2 card (bioMérieux, Marcy l’Etoile, France) (acceptable T identification index 0.22). Confirmation was done with a Microseq 500 16S ribosomal DNA bacterial kit (PE Applied Biosystems, Foster City, CA, USA). A 475-bp fragment was sequenced in an automated sequencer (360 ABI Prism, PE Applied Biosystems). The fragment was compared with National Center for Biotechnology Information (Bethesda, MD, USA) GenBank entries and showed 99% homology with *V. metschnikovii* (GenBank accession no. X74712.1). In vitro susceptibility testing with the AST-N032 Vitek 2 card (bioMérieux) showed that the organism was resistant to ampicillin, ticarcillin, piperacillin, and aminoglycosides.

The patient was successfully extubated. He was transferred to a pneumology ward on day 9 and discharged on day 15. Antimicrobial treatment was stopped on day 10.

Most nonhuman strains of *V. metschnikovii* are usually found in aquatic habitats (e.g., lakes and marine waters). Human clinical infections with this bacterium are rare; however, cases of epidemic diarrhea caused by *V. metschnikovii* have been reported (*5**,**6*). Contamination by water or fish was not always demonstrated in these cases, but an orofecal source is possible. In coproculture, this microorganism is probably not diagnosed: it was initially identified as normal aerobic flora because it was oxidase negative.

The first case of septicemia with *V. metschnikovii* was reported in 1981 in a patient with peritonitis and an inflamed gallbladder (*1*). Three other patients with similar septicemia, all >70 years of age, were described (*7**,**8*); 2 had polymicrobial results in blood cultures. *V. metschnikovii* was also found in a mucocutaneous site (wound infection) after saphenectomy in swab samples of the wound site (*9*).

The patient in our study denied contact with lake or sea water, and he had not eaten any seafood. He was a retired carpenter without contact with domestic or wild animals and did not recall an episode of diarrhea before his hospitalization. The source of contamination that caused his acute respiratory failure was not identified.

Miyake et al. showed that *V. metschnikovii* produces a cytolysin with hemolytic properties (*10*). This finding might explain the invasive process of this bacterium, which resulted in pulmonary lesions in a patient with respiratory deficiency. As far as we know, this is the first case of pneumonia caused by *V. metschnikovii*.
